# *OsINV3* and Its Homolog, *OsINV2*, Control Grain Size in Rice

**DOI:** 10.3390/ijms21062199

**Published:** 2020-03-23

**Authors:** Xiaoshu Deng, Xiaohang Han, Shicong Yu, Zhijian Liu, Daiming Guo, Yao He, Wenyi Li, Yu Tao, Chaowei Sun, Peizhou Xu, Yongxiang Liao, Xiaoqiong Chen, Hongyu Zhang, Xianjun Wu

**Affiliations:** 1 Rice Research Institute, Sichuan Agricultural University, Chengdu 611130, China; dengxss520@163.com (X.D.); hanxhasd@163.com (X.H.); shicongyu@aliyun.com (S.Y.); 18302879527@163.com (Z.L.); daiming_guo@outlook.com (D.G.); heyao2017@gmail.com (Y.H.); liwenyi0921@163.com (W.L.); 18408211896@163.com (Y.T.); zavierstayhere@outlook.com (C.S.); xpzhxj@163.com (P.X.); liaoyongxiang123@163.com (Y.L.); xiaochenq777@126.com (X.C.); 2 Key Laboratory of Southwest Crop Genetic Resources and Genetic Improvement, Ministry of Education, Chengdu 611130, China

**Keywords:** rice (*Oryza sativa*), grain size, *OsINV3*, *OsINV2*, sucrose metabolism

## Abstract

Vacuolar invertase is involved in sugar metabolism and plays a crucial role in plant growth and development, thus regulating seed size. However, information linking vacuolar invertase and seed size in rice is limited. Here we characterized a small grain mutant *sg2* (grain size on chromosome 2) that showed a reduced in grain size and 1000-grain weight compared to the wild type. Map-based cloning and genetic complementation showed that *OsINV3* is responsible for the observed phenotype. Loss-of-function of *OsINV3* resulted in grains of smaller size when compared to the wild type, while overexpression showed increased grain size. We also obtained a T-DNA insertion mutant of *OsINV2*, which is a homolog of *OsINV3* and generated double knockout (*KO*) mutants of *OsINV2* and *OsINV3* using CRISPR/Cas9. Genetic data showed that *OsINV2*, that has no effect on grain size by itself, reduces grain length and width in the absence of *OsINV3*. Altered sugar content with increased sucrose and decreased hexose levels, as well as changes vacuolar invertase activities and starch constitution in *INV3^KO^*, *INV2^KO^*, *INV3^KO^INV2^KO^* mutants indicate that *OsINV2* and *OsINV3* affect sucrose metabolism in sink organs. In summary, we identified *OsINV3* as a positive regulator of grain size in rice, and while *OsINV2* has no function on grain size by itself. In the absence of *OsINV3*, it is possible to detect a role of *OsINV2* in the regulation of grain size. Both *OsINV3* and *OsINV2* are involved in sucrose metabolism, and thus regulate grain size. Our findings increase our understanding of the role of *OsINV3* and its homolog, *OsINV2*, in grain size development and also suggest a potential strategy to improve grain yield in rice.

## 1. Introduction

The Rice is an important cereal crop that feeds majority of the global population. There is an urgent need to increase the yield of rice to support the rapid growth of global population. Grain size is one of the most important factors that determines grain yield in rice. Thus, investigation of grain size-associated genes and elucidation of their functional mechanisms have great significance for further improvement of rice yield [1]. So far, several genes related to grain size have been documented. These genes are involved in regulating multiple signaling pathways [2,3,4,5,6], including the ubiquitination-mediated proteasomal degradation pathway, the mitogen-activated protein kinase (MAPK) signaling pathway, G-protein signaling, phytohormone signaling pathway and transcriptional regulatory factors. For example, the genes involved in ubiquitination-mediated proteasomal degradation pathway include *GW2* [7], *HGW* [8], *TUD1* [9], and *WTG1* [10]. The genes involved in the MAPK signaling pathway include *OsMKK4* [11], *OsMAPK6* [12], *OsMKP1/GSN1* [13], and *OsMKKK10* [14,15]. The *OsMKKK10-OsMKK4-OsMAPK6* signaling pathway positively regulates grain size and weight in rice. Rice encodes a Gα, a Gβ, and five Gγ proteins [16]. The Gβ protein is essential for plant survival and growth, Gα provides a foundation for grain size expansion, while the Gγ proteins function as antagonists to regulate grain size [17]. The phytohormone pathway genes include *OsSGL* [18,19], *GAD1* [20], *SRS3* [21], and *GDD1* [22]. Another important set of signaling pathway genes controlling grain size are transcriptional regulatory factors, which play a crucial role in rice development, especially during the course of grain growth. For example, *OsPIL15* [23], a helix–loop–helix transcription factor, regulates grain size by directly targeting a purine permease gene *OsPUP7*. Recently, *GL6*, a new *QTL*, that encodes a plant-specific PLATZ (plant AT-rich sequence- and zinc-binding) transcription factor affecting grain length and spikelet number was identified [24]. However, the molecular roles of some regulators in grain size control are yet unclear or controversial [6]. The genetic relationships among different regulators and the molecular interactions amongst different signaling pathways are largely unknown. Invertase (EC 3.2.1.26) is a key enzyme in carbohydrate metabolism that irreversibly hydrolyzes sucrose into glucose and fructose, both of which are basic energy sources, and act as signaling molecules for plant growth, crop yield and stress responses [25,26,27,28]. Based on subcellular location, invertases are classified into cell wall invertases (CWINs), vacuolar invertases (VINs) and cytoplasmic invertases (CINs) [29]. CWINs and VINs have optimal activity at an acidic pH (3.5–5.5), while CINs function optimally in an alkaline or neutral pH (6.8–8.0) [29]. Little is known about the function and regulation of CINs. CWINs play a key role in assimilate partitioning, thus regulating grain weight in crops [30,31]. VINs regulate cell expansion, osmotic pressure, sugar signals, sucrose accumulation, and sucrose concentration, especially during the expansion phases of sink organs [32]. VIN belongs to β-fructofuranosidases, a group of N-glycosylated proteins that contain a β-fructosidase motif (NDPN) and a cysteine catalytic domain (WECVD), both of which are essential for the catalytic activity of VIN [28]. In Arabidopsis, a gene encoding vacuolar invertase was reported to control the lengths of roots and hypocotyls, especially in the elongating zones of roots [33]. Vacuolar invertase activity was detected in the cell elongation zone of the seminal root in maize seedlings [34]. It has also been speculated that VIN contributes to fiber cell elongation in cotton [35]. In potato, VIN is the key determinant in reducing sugar accumulation during cold-induced sweetening [36,37]. VIN silencing was found to significantly reduce cold-induced sweetening in stored potato tubers, thus addressing a long-standing quality problem in French fries [38]. In rice, two VIN isogenes, *OsINV2* and *OsINV3*, have been identified [39]. *OsINV3* plays a role in determination of sink strength by regulation of assimilated partitioning, and affects grain size and yield by altering sugar metabolism in rice, including sugar composition, transport and starch accumulation [40,41]. Recently, *OsINV2* was identified as a functionally redundant vacuolar invertase isoform as mutating it did not show any significant changes in key agronomic and physiological traits [42]. Despite the wealth of documented knowledge about vacuolar invertases in rice, the genetic relationships of the two VIN genes and the molecular interactions between VINs and grain size are largely unknown.

To further understand the molecular mechanisms that determine grain size, we identified a few genes that affect grain size in rice. Here we report a new mutant allele of *OsINV3* in the *indica* background, named *SG2*, which encodes a vacuolar invertase involved in molecular sink strength determination in rice. Our work focused on the physiological role and function of VIN isogenes, *OsINV2* and *OsINV3*. Our results suggest that both *OsINV3* and *OsINV2* influence grain size by regulating sucrose metabolism and that both genes are important regulatory factors required for grain size development in rice.

## 2. Results

### 2.1. Characterization of the *sg2-1* and *sg2-2* Mutants

**Figure 1:**
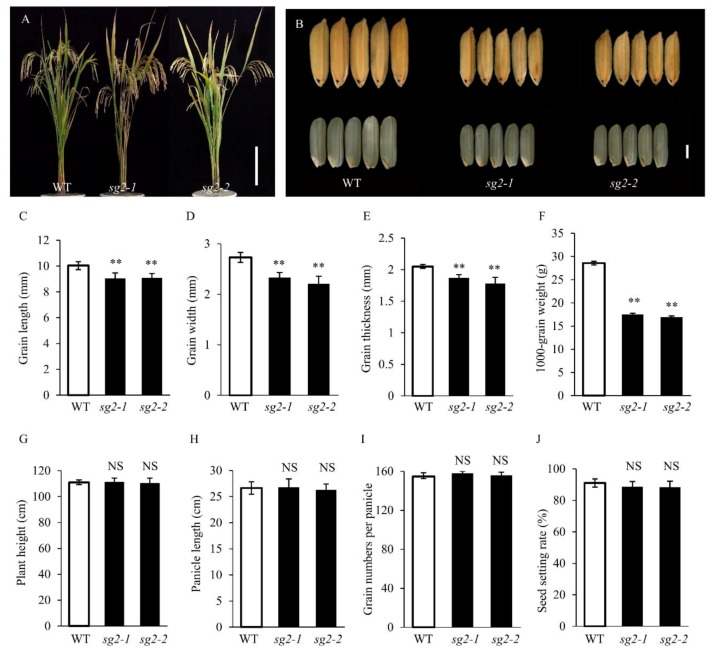
Phenotypic analysis of *sg2* mutants. (**A**) Plant comparison of wild-type (WT), *sg2-1*, *sg2-2* at the maturity stage. Bar = 20 cm. (**B**) Morphology of grain shape in WT, *sg2-1, sg2-2*. Bar = 2 mm. Statistical data of the grain length (**C**), grain width (**D**), grain thickness (**E**), 1000-grain weight (**F**), plant height (**G**), panicle length (**H**), grain numbers per panicle (**I**), and seed setting rate (**J**) in WT and *sg2* mutants. Data are given as means ± SD. Student’s t-test was used to generate the *p* values; ** and NS indicate *p* < 0.01 and no significant differences, respectively.

To understand how grain size is determined in rice, we selected two small seed mutants (*sg2-1* and *sg2-2*) from EMS-mutagenized M2 populations of Yixiang 1B (WT). When compared with WT, the *sg2-1* and *sg2-2* mutants exhibited a smaller grain phenotype (Figure 1A,B). Grain length of WT, *sg2-1* and *sg2-2* was 10.04 mm, 9.05 mm, and 9.08 mm, respectively (Figure 1C and Table 1). The *sg2-1* and *sg2-2* mutants showed reduced grain width and grain thickness (14.65% and 19.05% for grain width, 8.78% and 13.17% for grain thickness, respectively) compared to WT (Figure 1D,E and Table 1). Moreover, the 1000-grain weight of *sg2-1* and *sg2-2* was markedly reduced by 38.75%, 40.67% as compared to the WT (Figure 1F and Table 1). No obvious difference was observed in any other agronomic traits (Figure 1A,G–J and Table 1). These results indicate that *SG2* influences grain size and weight in rice.

### 2.2. *sg2* Regulates Spikelet Hull Development by Modulating Cell Expansion

**Figure 2:**
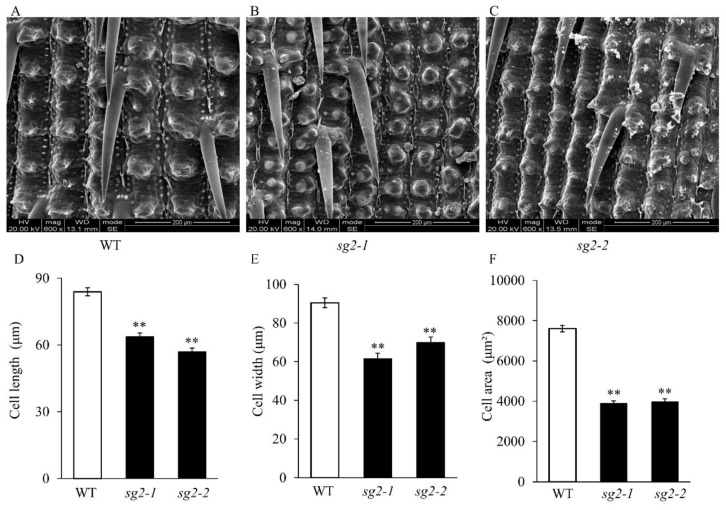
Histological comparison of the spikelet hulls between WT and *sg2* mutants. (**A**–**C**) Outer epidermal cells of the lemma observed by SEM. Scale bar, 200 μm. (**D**–**F**) Comparison analysis of the cell length, cell width and cell area in the outer epidermal cells. Data are given as means ± SD. Student’s t-test was used to generate the *p* values; ** indicate *p* < 0.01.

The size of a grain has been known to be restricted by its spikelet hull, which may set an upper limit for final grain size [4]. The growth of spikelet hulls is coordinately determined by cell proliferation and expansion. We therefore examined outer epidermal cells in spikelet hulls in WT, *sg2-1* and *sg2-2* by scanning electron microscopy (SEM). As shown in Figure 2A-C, the cell densities in *sg2-1* and *sg2-2* were significantly higher than that of WT, the cells were closely stacked, and the cell size was smaller. The cell length, cell width and cell area in *sg2-1* and *sg2-2* were significantly decreased (Figure 2D–F and Table S1) in agreement with the observations by SEM. Cell proliferation and cell expansion processes have been known to coordinately regulate spikelet hull growth [5]. We investigated expression of several known genes that determine grain size genes and are involved in the regulation of cell expansion, such as *GS2* [43], *GL7* [44], *SRS5* [45], *SRS3* [21], and *SMG11* [46]. We further investigated the expression levels of some known genes responsible for grain size involved in the regulation of cell proliferation, including *GS5* [47], *GS3* [48], *GW2* [7], and *GL3* [49]. The cell expansion genes were up-regulated in young panicles of *sg2-1* and *sg2-2* mutants, while the cell proliferation genes showed no difference in expression when compared to WT (Figure S1). Taken together, these results suggest that the small grain phenotypes of the *sg2-1* and *sg2-2* mutants are mainly a result of reduced cell expansion in spikelet hulls.

### 2.3. Genetic Analysis and Map-Based Cloning of the *sg2-1* and *sg2-2* Mutants

To identify the gene responsible for the *sg2* phenotype, we obtained F_1_ and F_2_ progenies from the crosses between mutants and WT. We found that the morphology of all F_1_ plants was similar to WT. In each of the F_2_ populations, the ratio of normal (WT) to small grains (*sg2* mutants) was around 3:1 (Table S2). Since *sg2-1* mutant had phenotypes very similar to *sg2-2*, we suspected that *sg2-1* and *sg2-2* are allelic mutants. The reciprocal crosses of *sg2-1* and *sg2-2* showed progeny with mutant phenotypes (Figure S2A). The grain size of *sg2-1/sg2-2* and *sg2-2/sg2-1* F_1_ progeny did not differ significantly from the *sg2* mutants (Figure S2B). Additionally, the grain length, grain width, grain thickness and 1000-grain weight of *sg2-1/sg2-2 and sg2-2/sg2-1* of F_1_ progeny were not significantly different when compared to the *sg2* mutants (Figure S2C–F). Overall, genetic analyses showed that *sg2-1* and *sg2-2* are controlled by a single recessive nuclear gene and are alleles.

**Figure 3:**
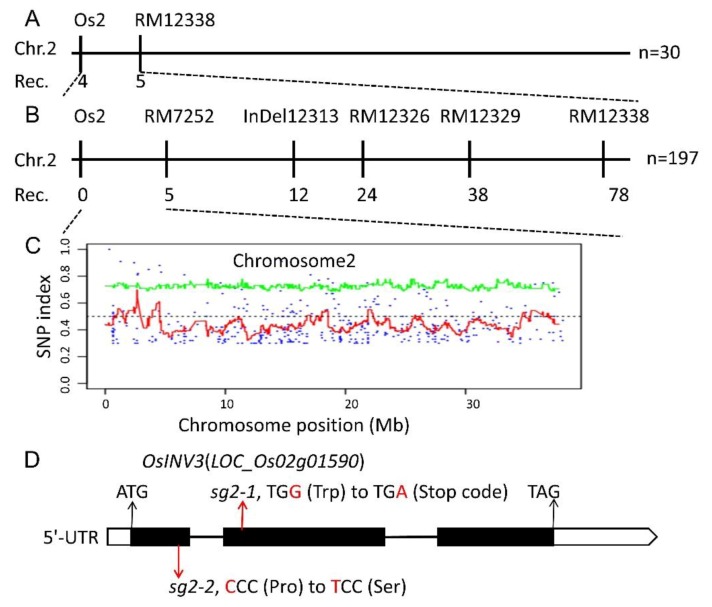
Positional cloning of *SG2*. (**A**) The *SG2* gene is located on chromosome 2 between InDel marker Os2 and SSR marker RM12338. (**B**) Then *SG2* gene is delimited to the region between Os2 and RM7252 using 197 F_2_ mutant individuals. (**C**) Manhattan plot of chromosome 2. (**D**) The gene structure of *SG2*. Black boxes, lines and white boxes represent exons, introns and the untranslated regions, respectively. The start codon (ATG) and the stop codon (TAG) are indicated. The *sg2* mutations in the *SG2* gene is shown.

The *sg2* mutations were initially mapped to a region between markers Os2 and RM12338 on chromosome 2 using the above-described F_2_ populations (Figure 3A) and were further narrowed down to a 130-kb genomic DNA region between Os2 and RM7252 (Figure 3B). Sequence comparison showed several SNPs between WT plants and the mutant pool. A single linked SNP (SNP-index = 1) was found by comparing sequences between the pooled mutants and WT in the candidate region (Figure 3C). The SNP was localized to the second exon of *LOC_Os02g01590* (Figure 3D). A G-to-A single base substitution was detected at the 878th base in *sg2-1*, that resulted in a premature stop codon. To define the molecular characteristics of the *sg2-2* allele, the *sg2-2* allele was amplified from genomic DNA by PCR and sequenced. Comparison of the sequences of WT and *sg2-2* revealed that *sg2-2* has a C to T substitution at base 421, resulting in a Proline (P) to Serine (S) change at amino acid 141. These results suggested that *LOC_Os02g01590* represents the *SG2* gene. This gene encodes a vacuolar invertase, *OsINV3*, which is involved in sink strength determination, mainly by regulation of grain size, assimilates partitioning to grain and affects grain size by altering sugar metabolism [40,41].

### 2.4. Confirmation of the *sg2/OsINV3* Gene

**Figure 4:**
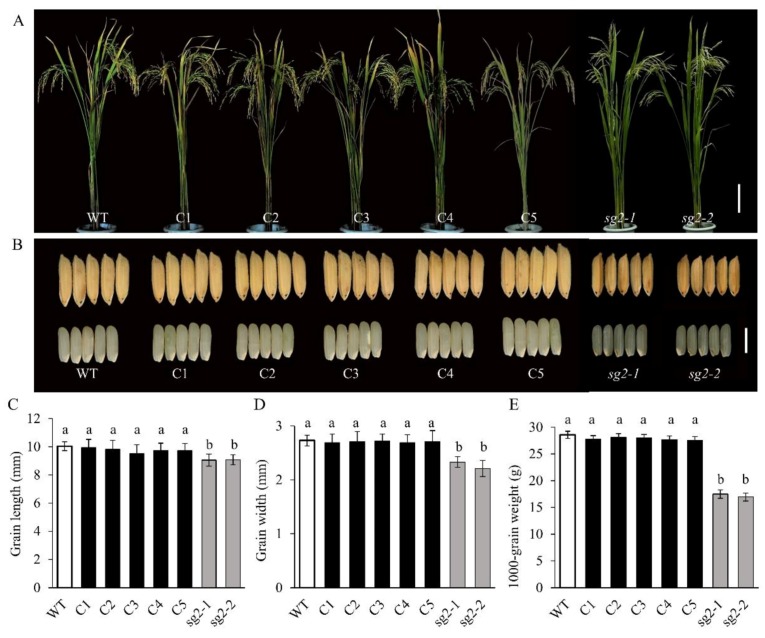
Phenotypic characterizations of complementation lines. (**A**) Plant comparison of wild-type (WT), complementation lines at the maturity stage. C1–C5 represent the five complementation transgenic lines. Bar = 20 cm. (**B**) Morphology of grain shape in WT, complementation lines. Bar = 2 mm. (**C**–**E**) Statistical data of the grain length (**C**), grain width (**D**), 1000-grain weight (**E**) in WT and complementation lines. Data are given as means ± SD. Different letters indicate statistically significant differences at the *p* = 0.01 level by Student’s t-test.

**Figure 5:**
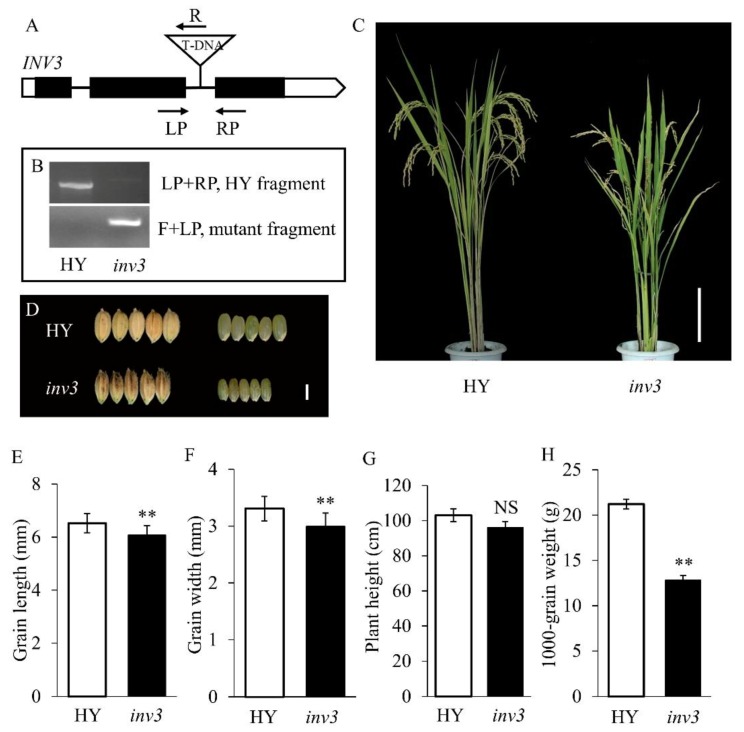
Phenotypic characterizations of *INV3* T-DNA insertion mutant. (**A**) T-DNA insertion site of the *inv3* mutant. The triangle indicates the T-DNA insertion site in the *inv3* mutant. Black boxes, lines and white boxes represent exons, introns and the untranslated regions, respectively. (**B**) Gel data showing the presence of HY and *inv3* alleles. (**C**) Plant comparison of HY, *inv3* mutant at the maturity stage. Bar = 10 cm. (**D**) Morphology of grain shape in HY, *inv3* mutant. Bar = 2 mm. (**E**–**H**) Statistical data of the grain length (**E**), grain width (**F**), plant height (**G**) 1000-grain weight (**H**) in WT and *inv3 mutant*. Data are given as means ± SD. Student’s t-test was used to generate the *p* values; ** and NS indicate *p* < 0.01 and no significant differences, respectively.

**Figure 6:**
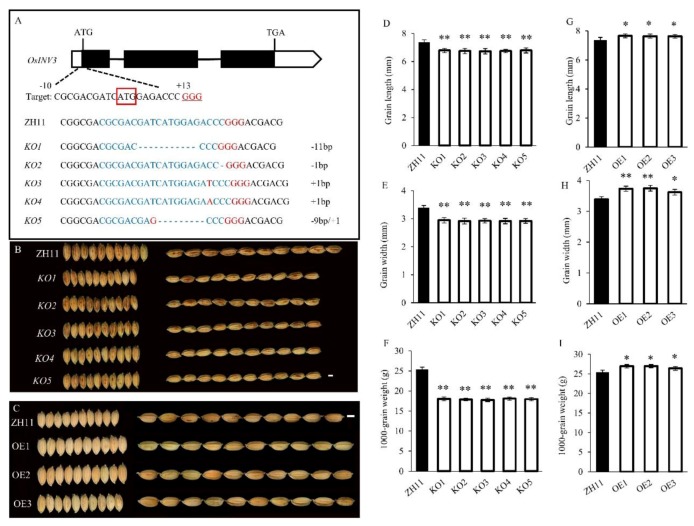
Genetic effects of *OsINV3 KOs* and OEs on grain size. (**A**) Schematic map of the sgRNA target site in *OsINV3* and sequence alignment for KOs (KO1–KO5). Black boxes, lines and white boxes represent exons, introns and the untranslated regions, respectively. The start codon (ATG) and the stop codon (TAG) are indicated. (**B**) Morphology of grain shape in ZH11 and *KOs*. Bar = 2 mm. (**C**) Morphology of grain shape in ZH11 and OEs (OE1–OE3). Bar = 2 mm. (**D**–**F**) Statistical data of the grain length (**D**), grain width (**E**), 1000-grain weight (**F**) in ZH11, *KOs*. (**G**–**I**) Statistical data of the grain length (**G**), grain width (**H**), 1000-grain weight (**I**) in ZH11, OEs. Data are given as means ± SD. Student’s t-test was used to generate the *p* values; **, * indicate *p* < 0.01, *p* < 0.05, respectively.

To confirm that *sg2* was a mutation in *OsINV3*, we performed a complementation experiment in the *sg2-1* background. A plasmid carrying wild-type gene of *LOC_Os02g01590* driven by its native promoter (*proINV3::INV3*) was introduced into the *sg2-1* mutant. Five complementation transgenic lines (C1-C5) were obtained (Figure 4A). All transgenic lines complemented the *sg2* phenotype (Figure 4B). The grain length, grain width and 1000-grain weight of C1, C2, C3, C4, and C5 did not differ as compared to WT (Figure 4C–E). The relative expression of *OsINV3* in the *sg2* mutants was significantly reduced in comparison to the WT, and the relative expression in complementation lines (C1-C5) reverted back to WT levels (Figure S3A). In addition, a T-DNA insertion mutant, *inv3*, was obtained in the background of Hwayoung (HY), a *japonica* variety. In *inv3*, the T-DNA was inserted between the second and third exons (Figure 5A,B). Compared to HY, the *inv3* T-DNA insertion mutant displayed reduced plant height, smaller grain size, and drastically reduced 1000-grain weight (Figure 5C–H, Table S3). We also generated knockout mutants (KOs) of *OsINV3* using the CRISPR/Cas9 genome editing system in the background of Zhonghua11 (ZH11) (Figure 6A). Five independent homozygous transgenic plants (*KO1*-*KO5*) with different mutations were generated that showed smaller grain size (Figure 6B,D–F, Table S4). The relative expression of *OsINV3* in the KOs was significantly decreased compared to ZH11 (Figure S3B). Three overexpression transgenic lines (OEs) were also obtained in ZH11 and their grain phenotypes were characterized (Figure 6C). Comparable to ZH11, the overexpression transgenic lines (OE1, OE2, and OE3) showed significant increases in grain length, width and weight (Figure 6G–I and Table S4). The relative expression of *OsINV3* in the OEs was significantly increased compared to ZH11 (Figure S3B). These results together confirmed that *LOC_Os02g01590* is indeed the *SG2* gene.

### 2.5. *OsINV3* Expression Pattern and Protein Subcellular Localization

**Figure 7:**
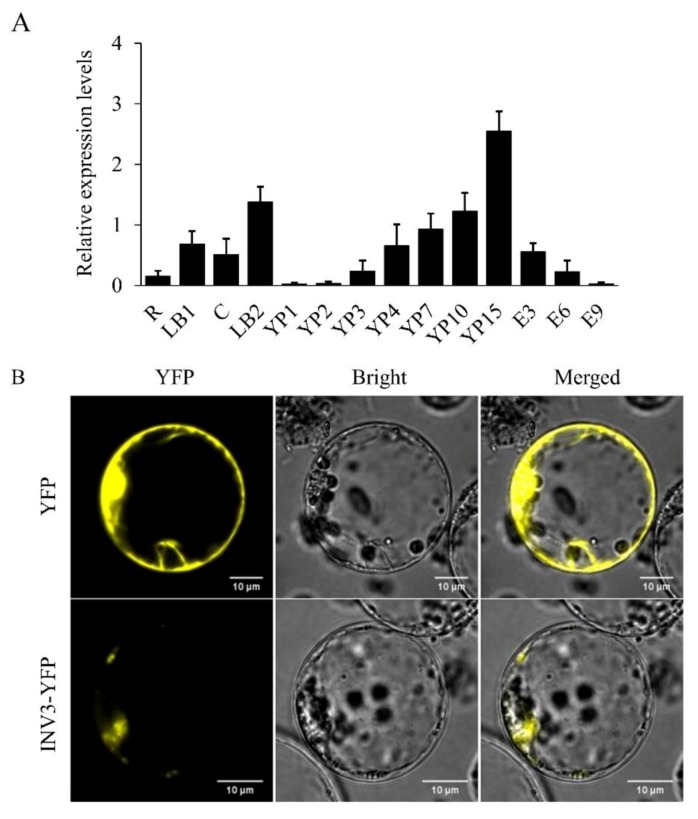
*OsINV3/SG2* expression pattern and protein subcellular localization. (**A**) Quantitative real-time PCR analysis of *OsINV3* expression in various tissues of wild-type plants. R, LB1, root and leaf blade at seedling stage; C, LB2, LS, culm, leaf blade, leaf sheath at booting stage, respectively; YP1-YP15, young panicles with different length (cm). E3-E9, endosperm, the number indicates the days after fertilization. Data are given as mean ±SD. (*n* = 3). *OsActin* was used as the control. (**B**) Subcellular localization of OsINV3 observed in rice protoplasts. Scale bar, 10 μm.

The expression pattern of *OsINV3* in various organs was investigated by quantitative real-time PCR. *OsINV3* transcripts were detected in all tissues tested, with significantly higher levels in the developing young panicles and leaf blade at booting stage, but at extremely lower levels in the developing endosperm (Figure 7A). We further investigated the subcellular localization of the OsINV3 protein. A plasmid containing the yellow fluorescent protein (YFP) gene tagged to *OsINV3* wild-type cDNA driven by a native promoter (*INV3-YFP*) was transiently expressed in rice protoplast. The INV3-YFP fusion protein localized to the nucleus (Figure 7B), an observation consistent with a previous report [41]. OsVIN2 has been reported to be a dual localization protein, and it is not detectable in the vacuole because the fluorescence signal is easily degraded in the acidic vacuolar lumen in the light and may be transported to the vacuole through PVCs [41,50,51]. Thus, the expression pattern of *OsINV3* is consistent with the role of *OsINV3* in influencing grain and panicle size.

### 2.6. Interaction between *OsINV3* and *OsINV2* in Grain Size Regulation

**Figure 8:**
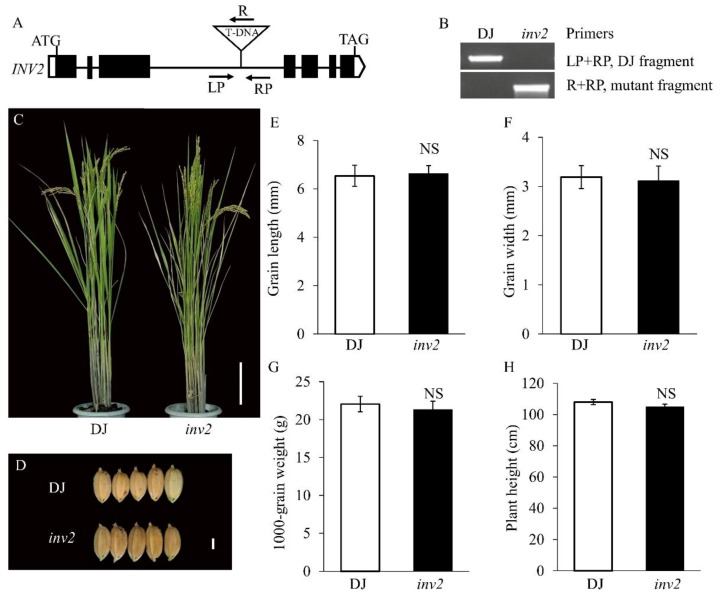
Phenotypic characterizations of *INV2* T-DNA insertion mutant. (**A**) T-DNA insertion site of the *inv2* mutant. The triangle indicates the T-DNA insertion site in the *inv2* mutant. Black boxes, lines and white boxes represent exons, introns and the untranslated regions, respectively. The start codon (ATG) and the stop codon (TAG) are indicated. (**B**) Gel data showing the presence of DJ and *inv2* alleles. (**C**) Plant comparison of DJ, *inv2* mutant at the maturity stage. Bar = 10 cm. (**D**) Morphology of grain shape in DJ, *inv2* mutant. Bar = 2 mm. (**E**–**H**) Statistical data of the grain length (**E**), grain width (**F**), plant height (**G**), and 1000-grain weight (**H**) in DJ and *inv2* mutant. Data are given as means ± SD. Student’s t-test was used to generate the *p* values; NS indicate no significant differences.

To further evaluate the underlying mechanisms of *OsINV3* and *OsINV2* in regulating grain size, we constructed a phylogenetic tree of all invertase proteins in rice. The analyses revealed a close phylogenetic relationship between OsINV2 and OsINV3 (Figure S4). We also obtained a T-DNA insertion mutant of *OsINV2* and generated double knockout (KO) mutants of *INVs* (*OsINV2* and *OsINV3*) using CRISPR/Cas9. The T-DNA insertion mutant *inv2* was obtained in the Dongjing (DJ) background, a *japonica* variety. In *inv2*, the T-DNA was inserted between the first and second exons (Figure 8A,B). When compared with DJ, *inv2* showed no visible differences in grain length, grain width or 1000-grain weight (Figure 8C–H and Table 2). For subcellular localization analysis of OsINV2, we generated an INV2-GFP fusion construct driven by the CaMV 35S promoter and performed a transient transfection assay in rice protoplasts. Fluorescent signals of INV2-GFP were detected in the nucleus, consistent with known OsINV3 localization (Figure S5). 

**Figure 9:**
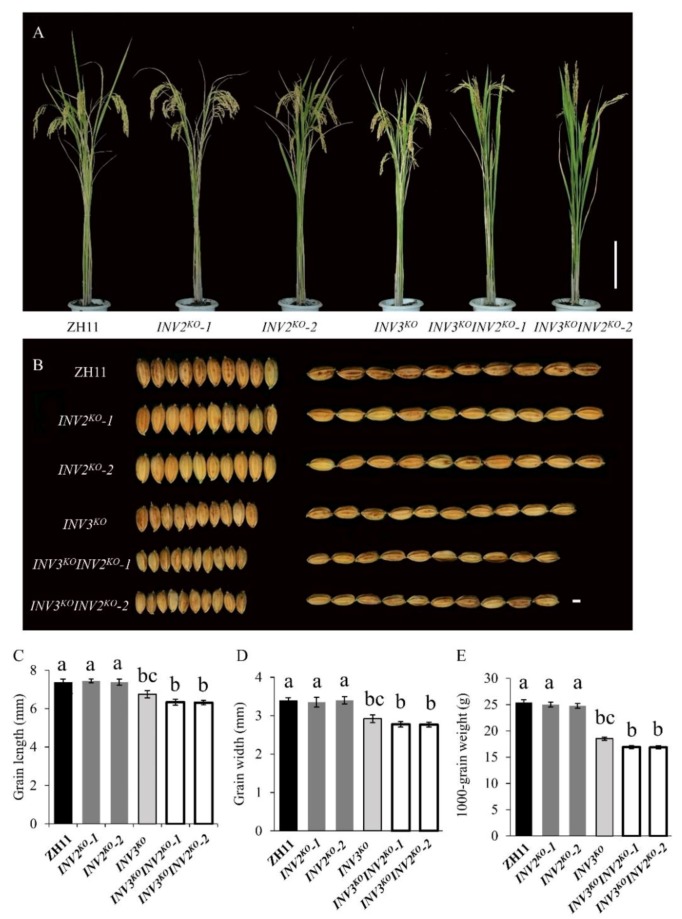
Genetic interactions of *INV2* and *INV3*. (**A**) Plant comparison of ZH11, *INV2^KO^-1*, *INV2^KO^-2*, *INV3^KO^*, *INV3^KO^INV2^KO^-1,* and *INV3^KO^INV2^KO^-2* at maturity stage. Bar = 25 cm. (**B**) Morphology of grain shape in ZH11, *INV2^KO^-1*, *INV2^KO^-2*, *INV3^KO^*, *INV3^KO^INV2^KO^-1,* and *INV3^KO^INV2^KO^-2*. Bar = 2 mm. (**C**–**E**) Statistical data of the grain length (**C**), grain width (**D**), 1000-grain weight (**E**) in ZH11, *INV2^KO^-1*, *INV2^KO^-2*, *INV3^KO^*, *INV3^KO^INV2^KO^-1,* and *INV3^KO^INV2^KO^-2*. Data are given as means ± SD. Different letters indicate statistically significant differences at the *p* = 0.01 level by Student’s t-test.

Additionally, we designed two sgRNA target sites (target 1 and target 2) in the first exon of *OsINV3* and third exon of *OsINV2* (Figure S6A). Interestingly, we obtained *OsINV2 KOs* (*INV2^KO^-1*, *INV2^KO^-2*), *OsINV3 KO* (*INV3^KO^*) and double knockout mutants (*INV3^KO^INV2^KO^-1*, *INV3^KO^INV2^KO^-2*) in the ZH11 background (Figure S6B). When compared with ZH11, the *OsINV2 KOs* showed the same grain size traits, but *INV3^KO^* and *INV3^KO^INV2^KO^* displayed obviously reduced grain size (Figure 9A,B). Compared to ZH11, *INV3^KO^*, *INV3^KO^INV2^KO^-1,* and *INV3^KO^INV2^KO^-2* showed dramatically reduced grain length and grain width, while *INV2^KO^-1* and *INV2^KO^-2* showed no difference (Figure 9C,D and Table 3). Moreover, the 1000-grain weight of *INV3^KO^*, *INV3^KO^INV2^KO^-1,* and *INV3^KO^INV2^KO^-2* was markedly reduced by 27.11%, 33.43%, and 33.56%, respectively as compared to ZH11, while the 1000-grain weight of *INV2^KO^-1* and *INV2^KO^-2* showed no difference (Figure 9E and Table 3). Compared with *INV3^KO^*, the two double mutants (*INV3^KO^INV2^KO^-1*, *INV3^KO^INV2^KO^-2*) showed markedly reduced grain width, grain length and 1000-grain weight, with grain length decreased by about 6.16% and 6.51%, and grain width decreased by about 5.00% and 5.31%, and 1000-grain weight decreased by about 8.68% and 8.86% respectively (Figure 9C–E and Table 3), suggesting that in the absence of *OsINV3*, it is possible to detect a role of *OsINV2* in the regulation of grain size. Furthermore, the relative expression of *OsINV3* and *OsINV2* in the double *KO* mutant was significantly reduced when compared to ZH11(Figure S7). 

To further explore the mechanisms controlling grain size, we examined outer epidermal cells in spikelet hulls in ZH11, *INV2^KO^*, *INV3^KO^*, and *INV3^KO^INV2^KO^* by SEM. In contrast to ZH11, the cell length, cell width and cell area of the *INV3^KO^* and *INV3^KO^INV2^KO^* were significantly reduced, while *INV2^KO^* showed no change in cell length, cell width and cell area (Figure S8). Collectively, these results suggest that *OsINV3* and *OsINV2* affect grain size, resulting in reduced grain size.

### 2.7. Analyses of the Physiological Role of VINs

**Figure 10:**
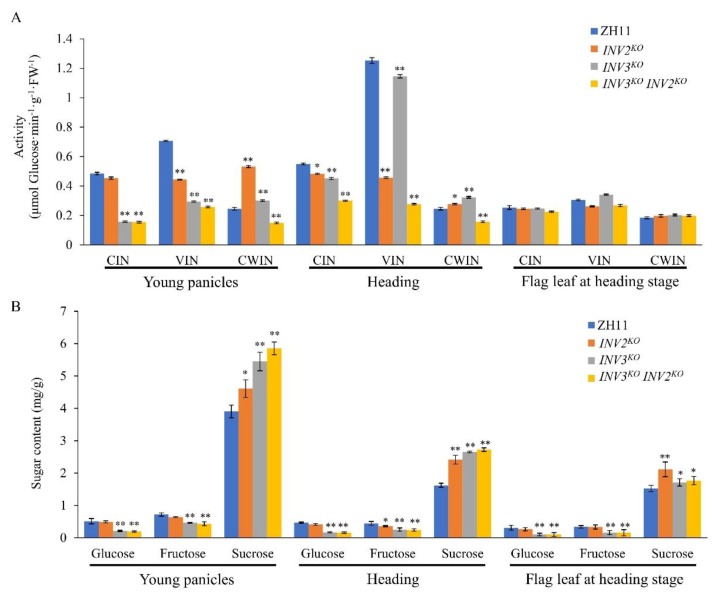
Comparisons of invertase activity and sugar content of ZH11, *INV2^KO^*, *INV3 ^KO^* and *INV3^KO^INV2^KO^*. Activities of three invertase isoforms (CIN, VIN, CWIN) (**A**) and sugar content (**B**) in young panicles, heading and flag leaf at heading stage of ZH11, *INV2^KO^*, *INV3^KO^*, and *INV3^KO^INV2^KO^*. Data are given as means ± SD. Student’s t-test was used to generate the *p* values; **, * indicate *p* < 0.01, *p* < 0.05, respectively.

As a major product of photosynthesis, sucrose is a key factor in crop yield. Vacuolar invertases are responsible for degradation of sucrose. To explore the physiological roles of *OsINV3* and *OsINV2* in determining grain size, we evaluated the invertase activities of CIN, VIN, CWIN, and measured the sugar composition of ZH11, *INV2^KO^*, *INV3^KO^*, and *INV3^KO^INV2^KO^* in young panicles, at heading stage and the flag leaf at heading stage. Compared to ZH11, the CWIN, VIN, and CIN activities of the *INV3^KO^INV2^KO^* were markedly reduced in young panicles and at heading stage, whereas the three invertase activities showed no difference in the flag leaf at heading stage (Figure 10A). The CIN and VIN activities were lower in *INV2^KO^* and *INV3^KO^* mutants, whereas the CWIN activities were higher in the ZH11 and VINs *KO* mutants (*INV2^KO^* and *INV3^KO^*) in young panicles and at heading stage (Figure 10A). Moreover, the invertase activities of the three isoforms in *INV2^KO^* and *INV3^KO^* mutants showed no difference in the flag leaf at heading stage (Figure 10A). The results of the analysis of sugar composition showed that glucose and fructose contents in young panicles and at heading stage (panicles and flag leaf) were lower in the *INV3^KO^* mutant and the *INV3^KO^INV2^KO^* double mutant when compared to the ZH11, whereas the *INV2^KO^* mutant showed no difference (Figure 10B). The sucrose content was higher in all *KO* mutants (*INV2^KO^*, *INV3^KO^*, and *INV3^KO^INV2^KO^*) when compared with ZH11 (Figure 10B). As reported previously, starch, which accounting for more than 70% of the final dry weight of mature grains, is synthesized from hexose units derived from sucrose [52]. To understand the influence of *OsINV2* and *OsINV3* on starch metabolism, we tested the starch contents of mature grains of *KOs*. The total starch content was not different between ZH11 and *KOs* grains, while the amylose content decreased by 3%–6% in grains from *KOs* (Figure S9). These results show that sugar composition and starch constitution are affected in the *INV2^KO^*, *INV3^KO^*, and *INV3^KO^INV2^KO^* mutants. This difference is owing to increased sucrose accumulation and reduced hexose production due to changes in invertase activities and starch constitution.

## 3. Discussion

### 3.1. *sg2* is a Novel Mutant Allele of *OsINV3*

Grain size mutants are ideal to investigate the molecular functions and regulatory mechanisms that determine grain size. In rice, several factors that control grain size have been identified, but the mechanisms remain largely unknown. In this study, we identified *SG2*, which encodes a vacuolar invertase, and is a novel allele of *OsINV3*, also known as *OsVIN2* [40,41]. The *sg2-1* and *sg2-2* mutants showed reduced grain length, grain width, and 1000-grain weight (Figure 1). Cell proliferation and cell expansion processes have been known to coordinately regulate spikelet hull growth [5]. Moreover, many studies have showed that VINs play key roles in plant growth by modulating cell expansion [33,53,54]. At the cellular level, the cell size of *sg2-1* and *sg2-2* mutants was affected by cell expansion in spikelet hulls (Figure 2), which is consistent with a previous report [41]. Map-based cloning and Mutmap analysis demonstrated that a SNP in the exon of *OsINV3* resulted in the mutant phenotype (Figure 3). Generally, VIN contains two key functional domains, NDPN and WECVD, which are essential for its catalytic activity [28]. A SNP mutation in *sg2-1* and *sg2-2* caused disruptions in the key function domains WECVD and NDPN, respectively, resulting in a nonfunctional invertase that eventually led to small seeds. The *sg2-1* and *sg2-2* showed phenotypes similar to the *inv3* alleles that have been shown to produce small grains by insertion mutations or by natural mutations [40,41]. Regardless of the nature of the mutation, the *OsINV3* mutants display small grain size, with *OsINV3* encoding a nonfunctional invertase. In agreement with previous studies, the *OsINV3* expression was found to be constitutive in different tissues (Figure 7). Furthermore, our results revealed that overexpression of *OsINV3* causes an increase in grain size, grain weight and grain yield (Figure 6C,G–I), indicating that this gene is a promising target for rice yield improvement. Together, these results suggest a novel and important role for *SG2/INV3* in grain size regulation.

### 3.2. Genetic Effect between *OsINV3* and *OsINV2* in the Regulation of Grain Size

Vacuolar invertases have been proposed to regulate cell expansion, osmotic pressure, sugar signals, sucrose accumulation, and sucrose concentration, especially during the expansion phases of sink organs [32]. In rice, two VIN isogenes, *OsINV2* and *OsINV3*, have been identified. The previously reported alleles of *OsINV3* have been shown to cause small grain size [40,41]. On the other hand, *OsINV2* was reported to be a functionally redundant vacuolar invertase isoform and showed no significant changes in key agronomic and physiological traits [42]. Both *OsINV3* and *OsINV2* play key roles in regulating traits related to grain yield in plant growth and development. Despite knowledge about these genes as outlined above, the genetic relationships of the two VIN genes and the molecular interactions between VINs and grain size are largely unknown. Moreover, there is no evidence to show any relationship between vacuolar invertase and seed size in rice, or how *OsINV2*, *OsINV3,* and *INV3-INV2* influence grain size. Our results show that *INV3^KO^* causes small grain size (Figure 5 and Figure 6), suggesting that *OsINV3* positively regulates grain size by causing cell expansion resulting in an increase in grain length and grain-width. Genetic data showed that *INV2^KO^* did not cause any change in grain size by itself (Figure 8 and Figure 9), but still plays important regulatory role in traits related to grain yield [42]. In our study, the double mutant *INV3^KO^INV2^KO^* showed a grain size smaller than both *INV3^KO^* and *INV2^KO^* (Figure 9), suggesting that in the absence of *OsINV3*, it is possible to detect a role of *OsINV2* in the regulation of grain size. As reported previously, the functions of *OsINV2* were redundantly encoded in *OsINV3* [42]. Our results show for the first time that *OsINV2* regulates grain size in the absence of *OsINV3*. Based on these findings, we predicted that both *OsINV3* and *OsINV2* have effects on grain size.

### 3.3. The Physiological Role of VINs in Regulating Grain Size

Grain biomass accumulation is dependent on sucrose supply and hexose assimilation along the transport pathway from leaf to developing caryopsis [25,27,28,32]. Sugar transporters play pivotal roles in carbon partitioning by mediating long distance sucrose transport from source leaves to sink [25]. Since invertases are responsible for the degradation of sucrose [32], we evaluated the physiological roles of *OsINV3* and *OsINV2* in regulating grain size, including the enzyme activity and sugar composition in ZH11 and *KO* mutants. When compared to ZH11, the VIN activities of all the *KO* mutants were reduced in young panicles and at heading stage, whereas there was no difference in activity among the three types of invertases in the flag leaf at heading stage (Figure 10A). The sugar composition was consistently affected in all *KO* mutants, resulting in increased sucrose accumulation and reduced hexose (glucose and fructose) production (Figure 10B). Interestingly, the invertase activities (CIN, VIN, CWIN) and sugar composition were found to be markedly reduced in the *INV3^KO^INV2^KO^* double mutant and the *INV3 ^KO^* single mutant when compared to ZH11. Starch is both an important caloric source and a molecule with a strong economic interest [55]. It is synthesized from hexose units derived from sucrose [52]. We also surveyed the starch contents of mature grains of *KOs*. There was no difference in the total starch content between ZH11 and *KOs* grains, while the amylose content decreased in grains from *KOs* (Figure S9). This indicated that sugar composition is affected in the single and double mutants, with increased sucrose accumulation and reduced hexose production as a consequence of changes in VIN, CIN activities, and starch constitution. The decreased invertase activity also resulted in reduced sucrose degradation, and grain biomass accumulation was blocked, resulting in smaller grain size.

## 4. Materials and Methods 

### 4.1. Plant Materials and Growth Conditions

The *sg2-1* and *sg2-2* mutants (small grain size gene on Chromosome 2, *sg2*) were isolated from an ethyl methane sulfonate (EMS)-mutagenized population of *indica* cultivar Yixiang1B (wild type, WT). The *Japonica* cultivars included 02428, Dongjing (DJ), Hwayoung (HY) and Zhonghua11 (ZH11). Rice seeds used in this study were sourced from WT plants, *INV3^KO^* transgenic plants, *INV2^KO^* transgenic plants, *OsINV3* overexpressing plants and T-DNA insertion mutants. Rice plants were planted in the fields at Wenjiang (Chengdu, China) in summer and Lingshui (Hainan, China) in winter. Seeds were sowed in fields and transplanted after 30 days. All materials were grown under natural environments and managed as breeding materials.

### 4.2. Agronomic Traits Analysis

For investigation of phenotypes, 15 randomly selected plants from a population were used for agronomic trait analysis at maturation stage. All phenotypic data were recorded from three biological replicates.

### 4.3. Scanning Electron Microscopy (SEM)

For SEM observation, spikelet hulls from WT, *sg2-1*, *sg2-2*, ZH11, and *KO* lines were collected before anthesis and fixed in 2.5% glutaraldehyde. The fixed samples were dehydrated in graded ethanol series. The samples were then dried in a critical-point drier, sputter-coated with gold, and observed by SEM (Inspect, FEI, USA) as previously described [56]. Cell length, cell width, and cell area were measured using Image J software.

### 4.4. Genetic Analysis and Map-Based Cloning

Mutmap [57] methodology was used for gene mapping. Briefly, four F_1_ and four F_2_ populations derived from the crosses WT×*sg2-1*, *sg2-1*×WT, WT×*sg2-2*, and *sg2-2*×WT were used for genetic analysis. F_2_ populations derived from the crosses 02428 × *sg2-1* and 02428 × *sg2-2* were used for mapping of the mutant gene. DNA from 25 BC_1_F_2_ plants with small grain phenotype similar to *sg2-1* was extracted and pooled in equal proportions, and subjected to whole-genome sequencing using Illumina Genome Analyzer IIx (Novogene, Beijing, China). Mixed DNA (5 μg) was used for preparation of libraries for Illumina sequencing according to the protocol for the Paired-End DNA Sample Prep kit (Novogene, Beijing, China). The libraries were used for cluster generation on a flow cell and sequenced for 76 cycles on an Illumina Genome Analyzer IIx. DNA from WT was re-sequenced as a control. The SNPs/INDELs indexes were calculated as previously described [57]. Sequences of the PCR primers used for mapping and the amplified *sg2-2* mutant genomic DNA sequence are given in Table S5.

### 4.5. Isolation of the *OsINV2* and *OsINV3* T-DNA Insertion Mutants

The T-DNA insertion mutants of *OsINV3* (stock no. PFG-2D-30640) and *OsINV2* (stock no. PFG-4A-50469) were obtained from the Korea Rice Mutant Database [58]. The *inv2* and *inv3* mutants were amplified using T-DNA primer R (5′-AACGCTGATCAATTCCACAG-3′) and primers specific to *OsINV2* and *OsINV3* genes. The primers used for genotyping were, LP (5′-TTGGCTGAGTGGTGGTGTC-3′) and RP (5′-GAGAGAGACACCAAATGATC-CATCC-3′) for *OsINV2*; LP (5′-CTTCCCTCCAGGTACACCTTC-3′) and RP (5′-GGAGGAGGAGAAGGGTTTTG-3′) for *OsINV3*.

### 4.6. Transgene Constructs

For complementation of the *sg2* mutation, a plasmid containing the full-length wild-type cDNA and 2000 bp upstream of the putative translation start site (*proINV3::INV3*) was constructed by cloning into the binary vector pCAMBIA1300-YFP and then introduced into the *sg2-1* mutant.

For overexpression, the 1986-bp CDS sequence of *OsINV3* from the WT was cloned under the control of the CaMV35S promoter into the binary vector pCAMBIA2300-GFP and then introduced into ZH11.

To obtain *KO* mutants, two sgRNA target sites of *OsINV3* (Figure 6A and Figure S6A) and one sgRNA target site of *OsINV2* (Figure S6A) were designed, and the CRISPR/Cas9 plasmid construct was generated using the methods described previously [59].

The primer sequences for construction of the above-described vectors are listed in Table S6.

### 4.7. Subcellular Localization of *OsINV3* and *OsINV2*

A plasmid containing full-length WT cDNA of *OsINV3* was cloned into the vector pCAMBIA1300 to generate an N-terminal fusion with the yellow fluorescent protein (YFP) under control of the *OsINV3* native promoter, resulting in pCAMBIA1300-INV3-YFP. Another plasmid containing the full-length CDS sequence of *OsINV2* from ZH11 was cloned under the control of the CaMV35S promoter into the binary vector pCAMBIA2300-GFP, resulting in pCAMBIA2300-INV2-GFP. Rice protoplasts were isolated from the leaves of WT seedlings (7-15 days after germination with dark treatment, 30 °C). pCAMBIA1300-INV3-YFP and pCAMBIA2300-INV2-GFP were introduced into the rice protoplasts using the method as described previously [60]. YFP or GFP fluorescence was detected using a confocal laser scanning microscope (Olympus FluoView FV1000, Japan).

### 4.8. Invertase Activity Assays and Determination of Sugar and Starch Contents

Young panicles (about 6~7 cm length panicles), heading stage and the flag leaf at heading stage were collected and assayed for invertase activity as described previously (VIN, CWIN, CIN) [40]. Samples for sugar content measurements were collected from young panicles (about 6~7 cm length panicles), heading stage and the flag leaf at heading stage and assayed as described previously [41]. The total starch content and amylose content in mature grains was measured using the Total Starch Assay Kit and the Amylose Assay Kit (https://www.cominbio.com), respectively, according to the manufacturer’s instructions.

### 4.9. RNA Isolation and RT-qPCR

Total RNA samples were obtained from the various plant tissues at different stages using the RNA Trizol (Invitrogen). One microgram of RNA was used to generate cDNA using a Revertase Transcription kit (Vazyme). The products were quantified using a real-time PCR detection system, following the manufacturer’s instructions (SYBR Green Master Mix, Vazyme). 

The PCR primers used are listed in Table S7. The rice Actin gene was used as an internal control.

## 5. Conclusions

The rice *sg2* mutant produces small grains. Map-based cloning revealed that the *SG2* gene, encoding a vacuolar invertase, is an allele of *OsINV3*. We identified that *OsINV3* is a positive regulator of grain size in rice while *OsINV2* has no effect on grain size by itself. However, in the absence of *OsINV3*, it is possible to detect a role of *OsINV2* in the regulation of grain size; the altered sugar composition, invertase activities and starch constitution in both single mutants (*INV3^KO^* and *INV2^KO^*) and double mutant (*INV3^KO^INV2^KO^*) lead to altered sucrose import and degradation capacities, regulating grain size.

## Figures and Tables

**Table 1:** Agronomic traits of WT and *sg2* mutants.

Materials	Grain Length (mm)	Grain Width (mm)	Grain Thickness (mm)	1000-Grain Weight (g)	Plant Height (cm)	Panicle Length (cm)	Grain Numbers per Panicle	Seed Setting Rate (%)
WT	10.04 ± 0.31	2.73 ± 0.10	2.05 ± 0.03	28.57 ± 0.36	111.00 ± 1.82	26.64 ± 1.21	154.70 ± 3.66	91.01 ± 2.61
*sg2-1*	9.05 ± 0.43 **	2.33 ± 0.10 **	1.87 ± 0.05 **	17.5 ± 0.29 **	111.40 ± 2.97	26.79 ± 1.59	158.00 ± 2.18	88.61 ± 3.29
*sg2-2*	9.08 ± 0.35 **	2.21 ± 0.15 **	1.78 ± 0.10 **	16.95 ± 0.25 **	110.50 ± 3.69	26.32 ± 1.10	156.00 ± 3.04	88.46 ± 3.77

Data are given as means ± SD. Student’s t-test was used to generate the *p* values; ** indicate *p* < 0.01.

**Table 2:** Agronomic traits of Dongjing (DJ) and *inv2.*

Materials	Grain Length (mm)	Grain Width (mm)	1000-Grain Weight (g)	Plant Height (cm)
DJ	6.53 ± 0.43	3.19 ± 0.24	22.04 ± 1.03	108.02 ± 1.62
*inv2*	6.63 ± 0.33	3.12 ± 0.30	21.32 ± 1.10	105.11 ± 1.52

Data are given as means ± SD. Student’s t-test was used to generate the *p* values.

**Table 3:** Agronomic traits of ZH11 and *KOs*.

Materials	Grain Length (mm)	Grain Width (mm)	1000-Grain Weight (g)
ZH11	7.36 ± 0.19	3.39 ± 0.09	25.41 ± 0.55
*INV2^KO^-1*	7.45 ± 0.10	3.35 ± 0.13	25.00 ± 0.48
*INV2^KO^-2*	7.38 ± 0.16	3.40 ± 0.10	24.76 ± 0.43
*INV3^KO^*	6.75 ± 0.18 **	2.92 ± 0.09 **	18.52 ± 0.31 **
*INV3^KO^INV2^KO^-1*	6.34 ± 0.15 **	2.78 ± 0.07 **	16.91 ± 0.29 **
*INV3^KO^INV2^KO^-2*	6.31 ± 0.11 **	2.77 ± 0.06 **	16.88 ± 0.30 **

Data are given as means ± SD. Student’s t-test was used to generate the *p* values; ** indicate *p* < 0.01.

## Supplementary Materials

Supplementary materials can be found at https://www.mdpi.com/1422-0067/21/6/2199/s1.

## Abbreviations

KO	Knockout
MAPK	Mitogen-Activated Protein Kinase
PLATZ	Plant AT-rich sequence- and zinc-binding
CWINs	Cell Wall Invertases
VINs	Vacuolar Invertases
CINs	Cytoplasmic Invertases
SEM	Scanning Electron Microscopy
SNPs	Single-Nucleotide Polymorphisms
PCR	Polymerase Chain Reaction
YFP	Yellow Fluorescent Protein
PVCs	Prevacuolar compartments
