# Supplementary material for: OsINV3 and Its Homolog, OsINV2, Control Grain Size in Rice
Source: Int J Mol Sci. 2020 Mar 23;21(6):2199. doi: 10.3390/ijms21062199 (PMC7139340; doi:10.3390/ijms21062199)
Supplement: Supplementary file 1 [file ijms-21-02199-s001.pdf]

International Journal of
Molecular Sciences

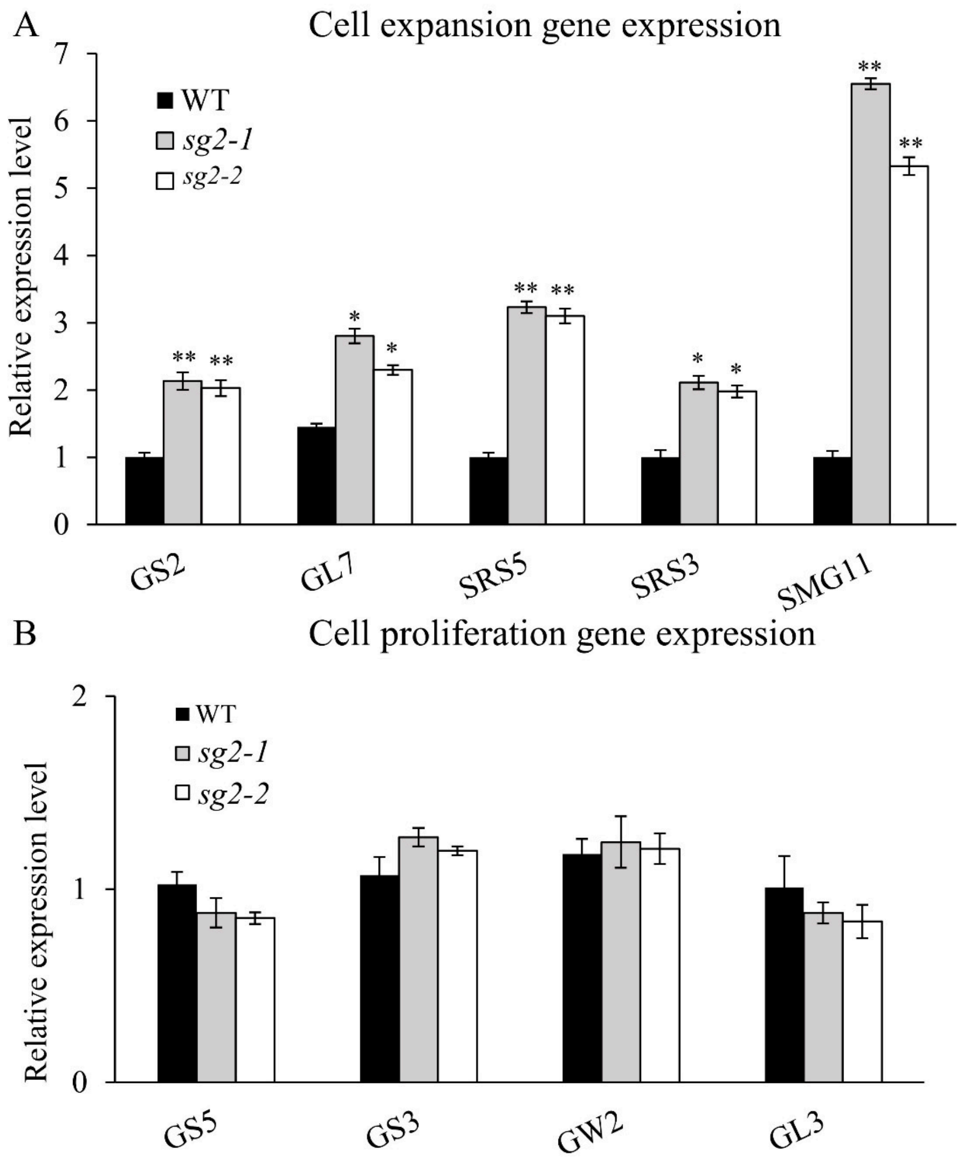

Figure S1. Transcript levels of the cell expansion genes (**A**) and cell proliferation genes (**B**) in young panicles (about 6~7 cm length panicles) of WT, *sg2-1* and *sg2-2*. *OsActin* was used as the control. Data are given as means ± SD. Student's t-test was used to generate the *p* values; **, * indicate $p < 0.01$, $p < 0.05$, respectively.

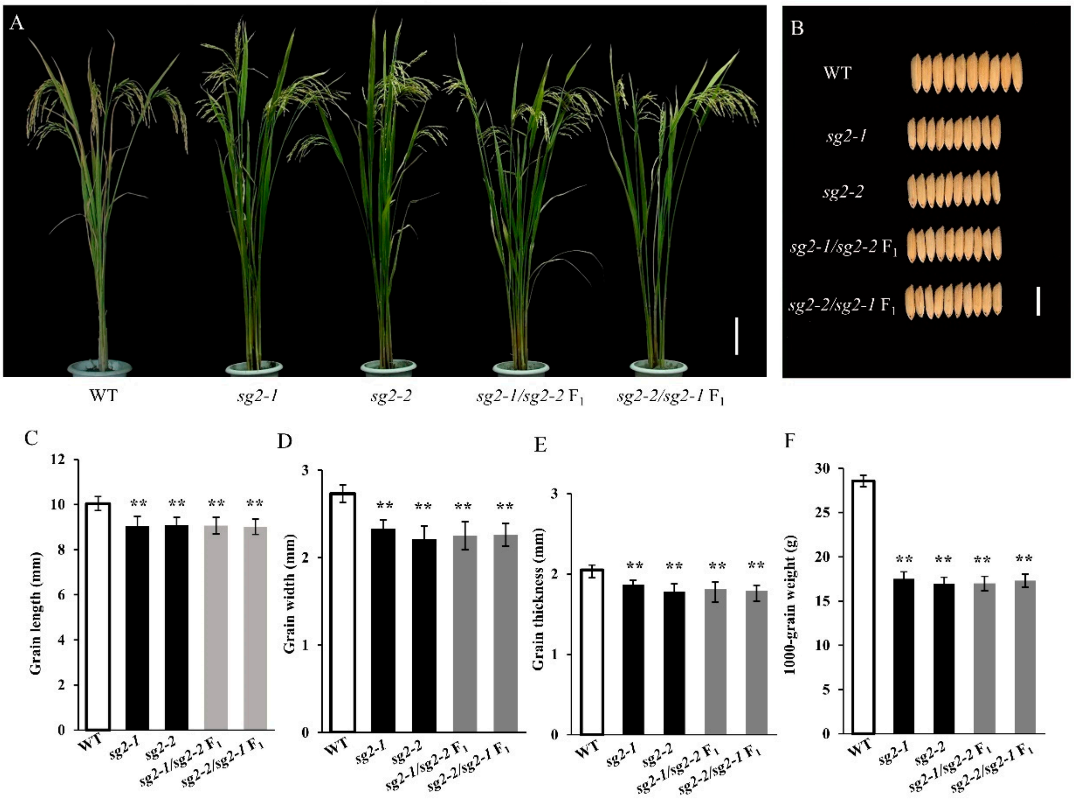

Figure S2. Allelic analysis between *sg2-1* and *sg2-2*. (**A**) Plant comparison of WT, *sg2-1*, *sg2-2*, *sg2-1*/*sg2-2* F_1 and *sg2-2*/*sg2-1* F_1 at the maturity stage. Bar = 20 cm. (**B**) Morphology of grain shape in WT, *sg2-1*, *sg2-2*, *sg2-1*/*sg2-2* F_1 and *sg2-2*/*sg2-1* F_1. Bar = 2 mm. Statistical data of the grain length (**C**), grain width (**D**), 1,000-grain weight (**E**), plant height (**F**) in WT, *sg2-1*, *sg2-2*, *sg2-1*/*sg2-2* F_1 and *sg2-2*/*sg2-1* F_1. Data are given as means ± SD. Student's t-test was used to generate the *p* values; ** indicate $p < 0.01$.

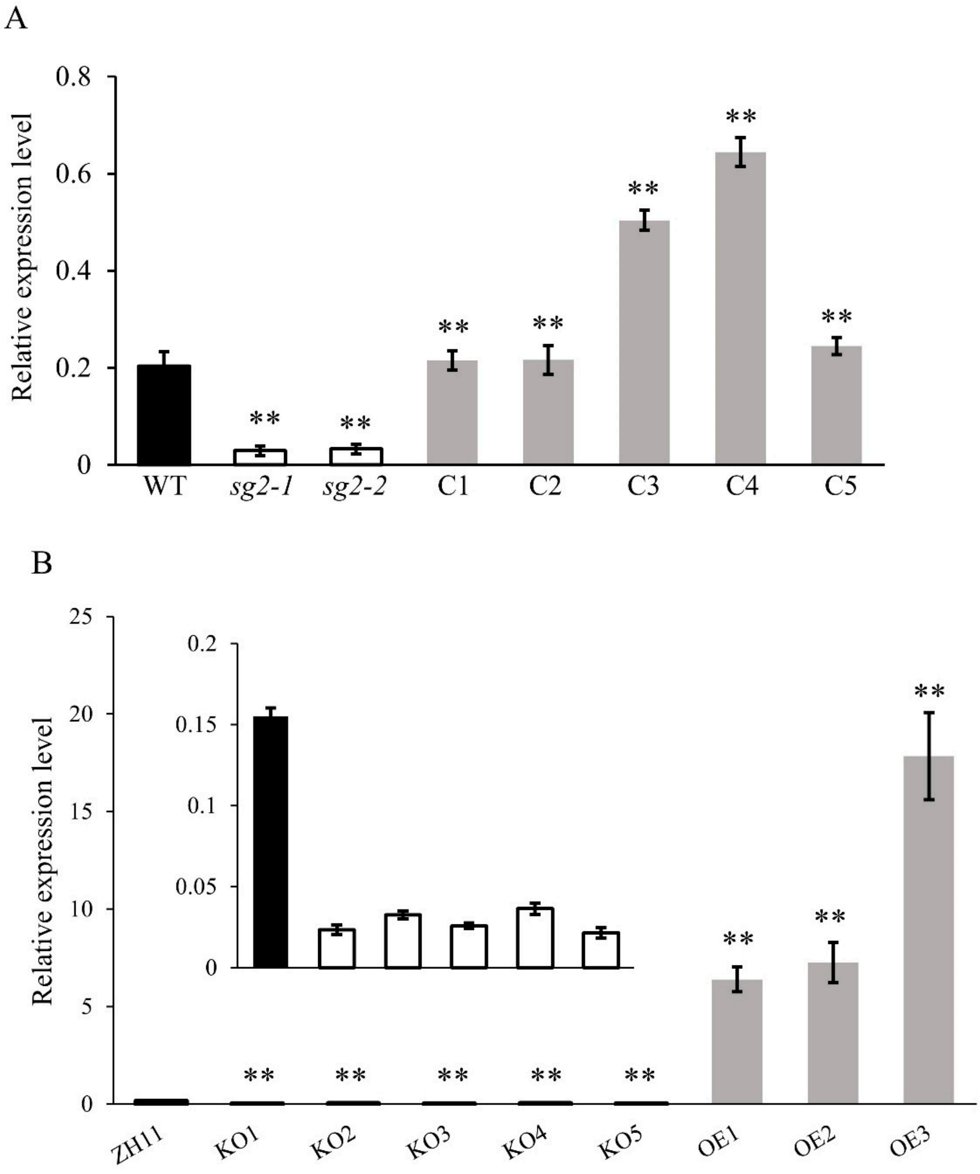

Figure S3. (**A**) Relative expression levels of *OsINV3* in young panicles (about 6~7 cm length panicles) of WT, *sg2-1*, *sg2-2* and complementation lines (C1-C5). (**B**) Relative expression levels of *OsINV3* in ZH11, *KOs* (*KO1-KO5*) and OEs (OE1-OE3). *OsActin* was used as the control. Data are given as means ± SD. Student's t-test was used to generate the p values; ** indicate $p < 0.01$.

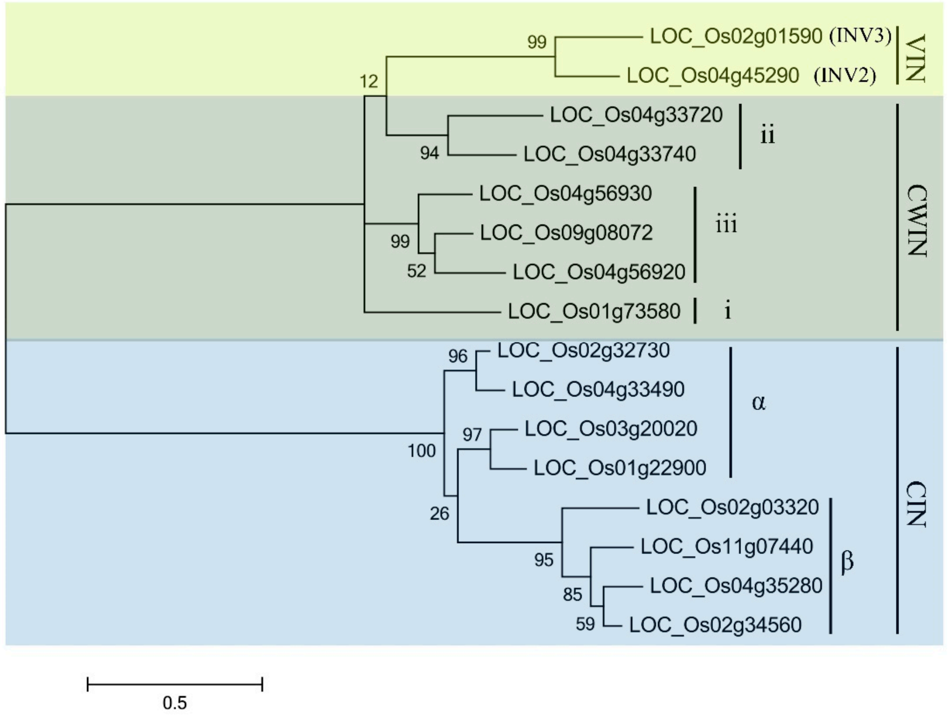

Figure S4. A phylogenetic tree of all invertase proteins of rice.

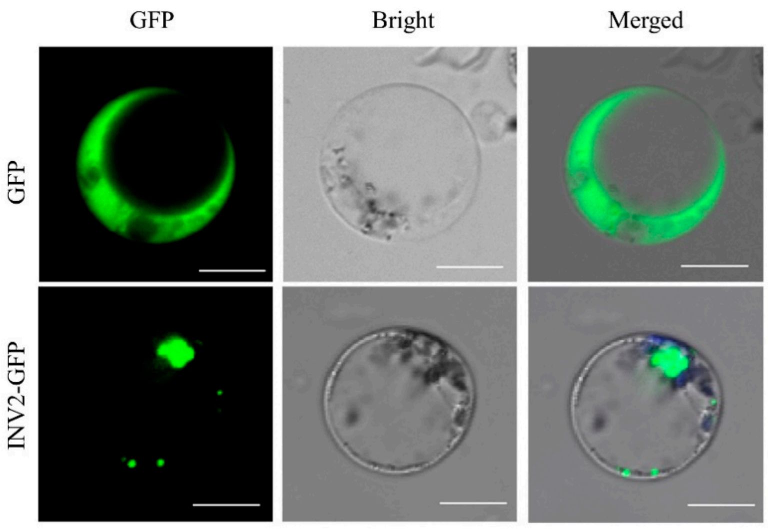

Figure S5. Subcellular localization of OsINV2 observed in rice protoplasts. Scale bar, 10μm.

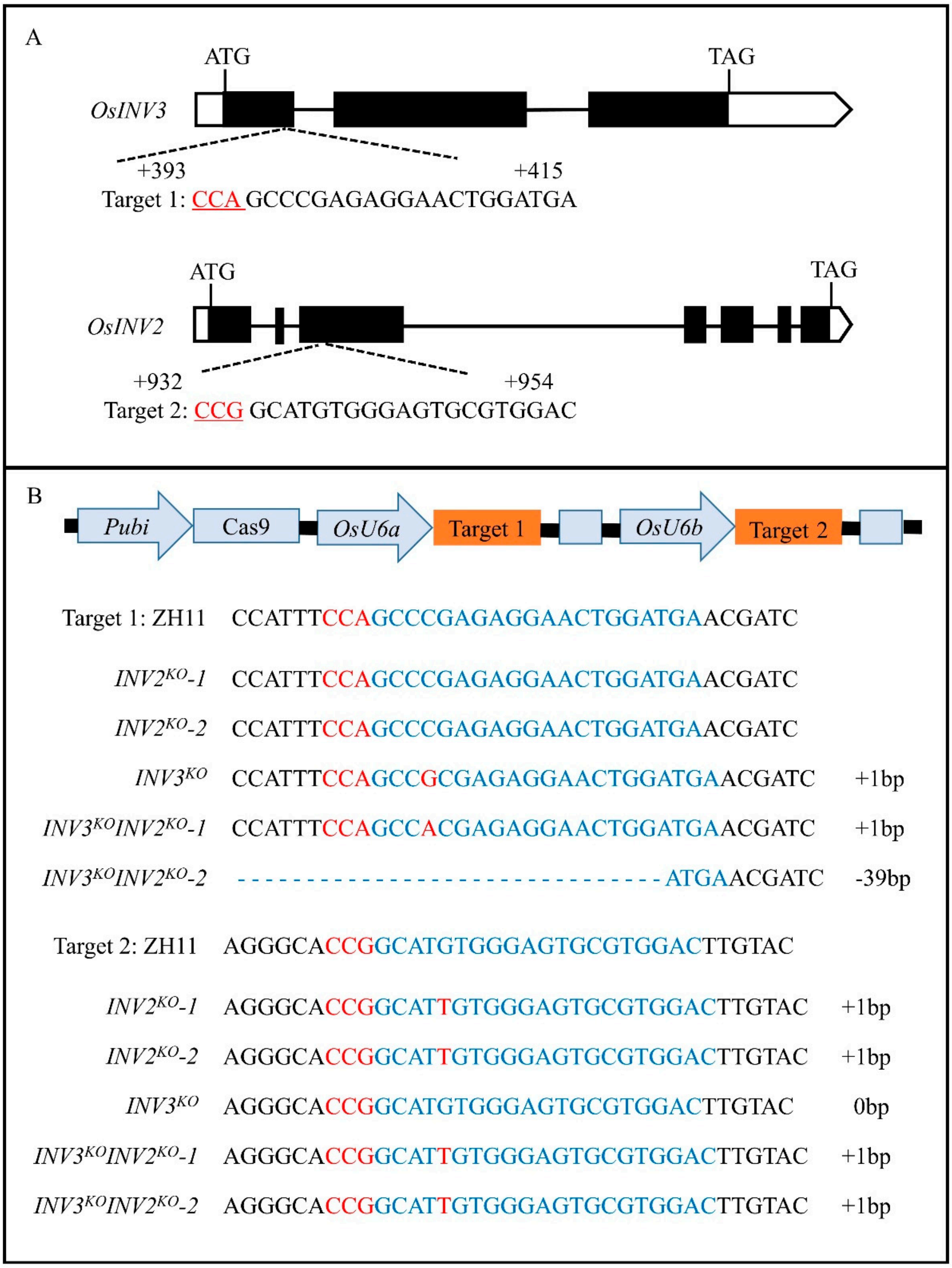

Figure S6. Knock-out mutants of created by CRISPR/Cas9. (**A**) Schematic map of the sgRNA target sites in *INV3* and *INV2*. Black boxes, lines and white boxes represent exons, introns and the untranslated regions, respectively. The start codon (ATG) and the stop codon (TAG) are indicated. (**B**) Vector construction and sequence alignment for $INV2^{KO}$ single mutants, $INV3^{KO}$ single mutant and $INV3^{KO}INV2^{KO}$ double mutants.

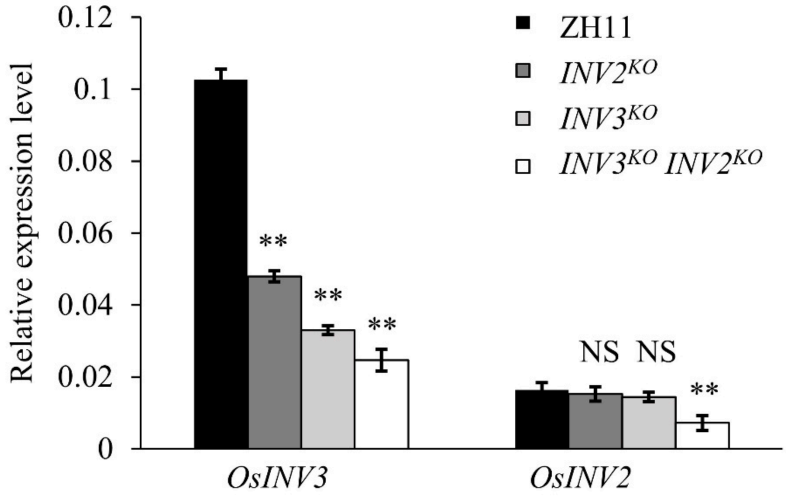

Figure S7. Relative expression levels of *OsINV3* and *OsINV2* in young panicles (about 6~7 cm length panicles) of ZH11, $INV2^{KO}$, $INV3^{KO}$ and $INV3^{KO}INV2^{KO}$. *OsActin* was used as the control. Data are given as means ± SD. Student's t-test was used to generate the *p* values; ** and NS indicate $p < 0.01$ and no significant differences, respectively.

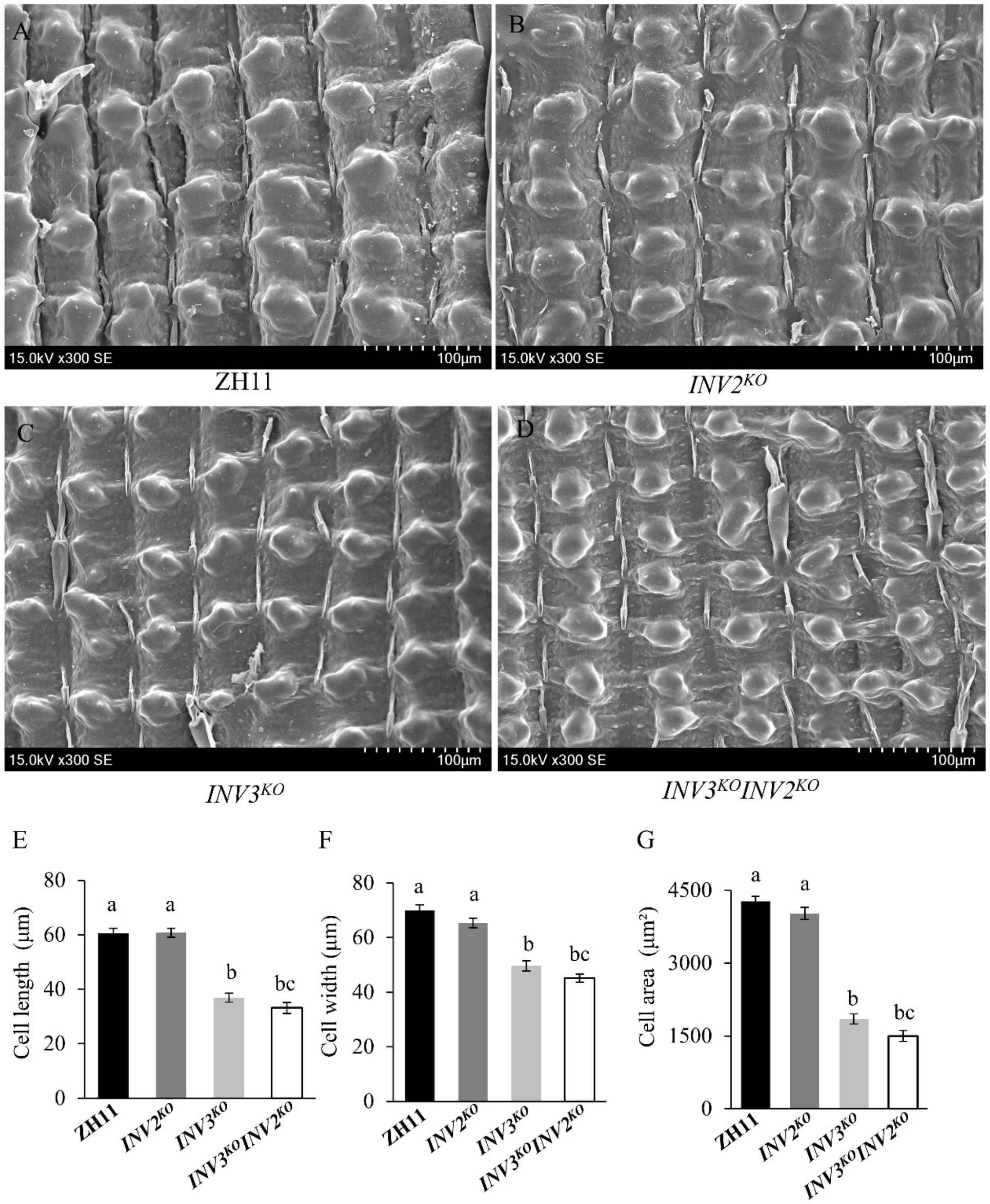

Figure S8. Histological comparison of the spikelet hulls in ZH11, *INV2*^{KO}, *INV3*^{KO} and *INV3*^{KO}*INV2*^{KO}. (**A**–**D**) Outer epidermal cells of the lemma observed by SEM. Scale bar, 100 μm. (**E**–**G**) Comparison analysis of the cell length, cell width and cell area in the outer epidermal cells. Data are given as means ± SD. Different letters indicate statistically significant differences at the p = 0.01 level by Student's t-test.

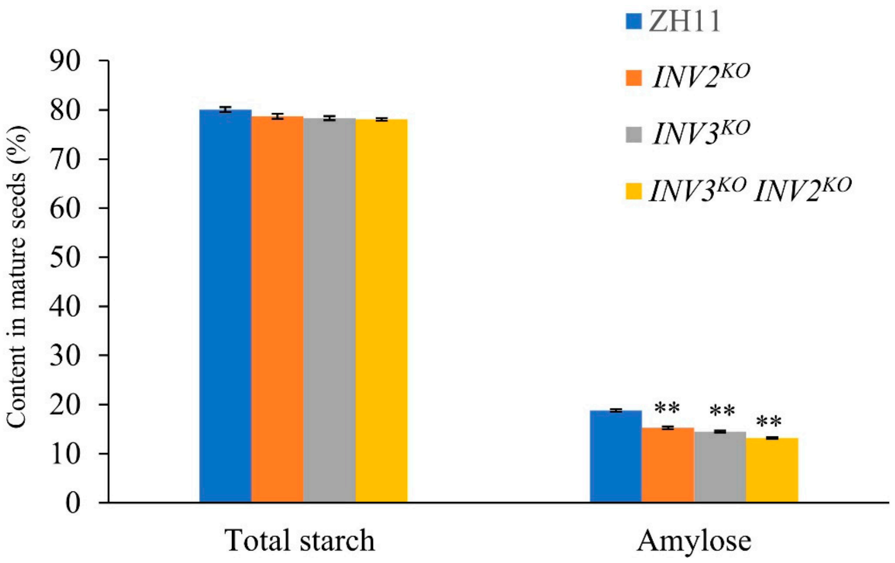

Figure S9. Total starch and amylose contents in mature grains of ZH11, *INV2*^{KO}, *INV3*^{KO} and *INV3*^{KO}*INV2*^{KO}. Data are given as means ± SD. Student's t-test was used to generate the *p* values; ** indicate $p < 0.01$.

Table S1. The lemma cell characteristics of WT, *sg2-1* and *sg2-2*.

	Cell Length (μm)	Cell Width (μm)	Cell Area (μm²)
WT	83.81 ± 1.83	90.45 ± 2.58	7605.62 ± 168.53
sg2-1	63.71 ± 1.69**	61.5 ± 2.83**	3915.87 ± 108.43**
sg2-2	56.90 ± 1.71**	69.97 ± 2.73**	3996.34 ± 110.53**

Data are given as means ± SD. Student's t-test was used to generate the *p* values; **indicate $p < 0.01$.

Table S2. Segregation ratio of F_2 populations.

Cross	NP	SP	Segregation ratio	$\chi^2_{0.05} < 3.84$	*p*
WT/*sg2-1*	451	141	3.20	0.38	$p < 0.05$
sg2-1/WT	621	199	3.12	0.23	$p < 0.05$
WT/sg2-2	427	134	3.19	0.46	$p < 0.05$
sg2-1/WT	602	197	3.06	0.04	$p < 0.05$

Note: NP, normal grain size phenotype plants. SP, small grain size phenotype plants. The segregation ratio was tested using the chi-square test.

Table S3. Agronomic traits of HY and *inv3* mutant.

	Grain Length (mm)	Grain Width (mm)	Plant Height (cm)	1000-Grain Weight (g)
HY	6.53 ± 0.33	3.31 ± 0.19	103.00 ± 3.63	21.21 ± 0.55
inv3	6.01 ± 0.32**	3.00 ± 0.23**	96.00 ± 3.58	12.81 ± 0.49**

Data are given as means ± SD. Student's t-test was used to generate the *p* values; **indicate $p < 0.01$.

Table S4. Agronomic traits of ZH11, *KOs*, OEs of *OsINV3*.

	Grain Length (mm)	Grain Width (mm)	1000-Grain Weight (g)
ZH11	7.36 ± 0.19	3.39 ± 0.09	25.41 ± 0.55

KO1	6.79 ± 0.15**	2.94 ± 0.09**	18.02 ± 0.49**
KO2	6.75 ± 0.19**	2.92 ± 0.10**	17.88 ± 0.35**
KO3	6.73 ± 0.19**	2.92 ± 0.08**	17.77 ± 0.42**
KO4	6.75 ± 0.12**	2.91 ± 0.09**	18.08 ± 0.39**
KO5	6.79 ± 0.18**	2.92 ± 0.08**	17.95 ± 0.41**
OE1	7.65 ± 0.13*	3.73 ± 0.08**	26.94 ± 0.43*
OE2	7.64 ± 0.14*	3.75 ± 0.09**	26.98 ± 0.42*
OE3	7.63 ± 0.13*	3.62 ± 0.09*	26.39 ± 0.48*

Data are given as means ± SD. Student's t-test was used to generate the p values; **, * indicate $p < 0.01$, $p < 0.05$, respectively.

Table S5. The List of polymorphic molecular markers for mapping and the *sg2-2* mutant genomic DNA sequence amplified.

Primer Name	Forward (5′-3′)	Reverse (5′-3′)
Os2	GATGACGGACGAAGAAATAC	TTTAAACCCAAAACATCGAG
RM12338	AGCTCAAGCTCAAGCTCACAACC	TGCACTGCAACCTAAACCTTTCC
RM7252	GGAGGAGGAGAAGGGTTTTG	ACGCGCTGTCAAGTTAAAGG
InDel12313	TTGGCTGAGTGGTGGTGTC	AGCTAGGAGCATCAACCCA
RM12326	GAGAGAGACACCAAATGATCCATCC	ACTGATTTGGCCCTTGTTCTTGG
RM12329	AGGAAGAGGCGAAGGTAGATCG	CCAATCATGCTGTGTTTCAAGG
sg2-2-exon1	CTGGCAACTGGGCCAAAT	CCATAAAACGTTCCTCCAAAAT
sg2-2-exon2	CGCTCTGTTCGTTCGTTCTT	CAATGGAGCCCAGTAAAAGTG
sg2-2-exon3	CATCCAGGAATGAATGCGGATA	TCTGAAGGCCAGGCCAGC

Table S6. The PCR primers used for vector construction.

Primer Name		Squence	Used for
1300-INV3-YFP	Forward	ggtacccggggatcctctagaGGAGCAGCTGGCTAATAAAATTAAA	Complementation
	Reverse	acgacggccagtgccaagcttTGGGGCTTGCATATTGATCTTG	
2300-INV3-GFP	Forward	atttggagaggacagggtaccATGGAGACCCGGGACGACG	Overexpression
	Reverse	agtgtcgactctagaggatccGGCCATGTAGGCTTGGTTGTA	
INV3^{KO}-U6a	Forward	gccgCGCGACGATCATGGAGACCC	CRISPR
	Reverse	aaacGGGTCTCCATGATCGTCGCG	
INV3^{KO}INV2^{KO}-U6a	Forward	gccgTCATCCAGTTCCTCTCGGGC	CRISPR
	Reverse	aaacGCCCGAGAGGAACTGGATGA	
INV3^{KO}INV2^{KO}-U6b	Forward	gttgTCCACGCACTCCCACATGC	CRISPR
	Reverse	aaacGCATGTGGGAGTGCGTGGA	

Table S7. List of primers for qRT-PCR.

Markers	Forward (5′-3′)	Reverse (5′-3′)
GS2	TGCGTCCCTTCTTTGATGAGT	ACAGTTGGGTGCCTGAGAATG
GL7	CCCCTAGCATCGACACCAAG	CGGGTTCCAGCACTCCTCT
SRS5	ATGAGGGAGTGCATCTCGAT	CAAGATCGACGAAGACAGCA
SRS3	CAAGATCGACGAAGACAGCA	CTGAGAAGCTGAAGCAGATG
SMG11	CCAACTGGAAGAGGAGAACATA	ACATGTAGTCTGTCCATTGCAA
GS3	CGGAAGAACTCCTGATCCATTC	CACTTGCTCTGCACAAACAGC
GW2	CAGCAGCGCATTCCCAGTTTTC	GTGGTCAGCCGAGCACTCTC
GS5	AGTGGACTGCTTCCAGGGAAG	CACGCAGTACCGAGAACTGA
GL3	GCTCAAGGTCACCTGATCACTC	GAACGACCACAAGATCTCTGC
OsINV3	CGGATTCCATTTCCAGCC	ACCACTGGTCGGGAACCA
OsINV2	GGCAGCTTGGTACCTGTGCTA	GCAGAGTTCAGCTCCCAAATC
Actin	ACCATTGGTGCTGAGCGTTT	CAGCTTCCATTCCTATGAA
